# Opportunistic diagnosis of osteoporotic vertebral fractures on standard imaging performed for alternative indications

**DOI:** 10.1259/bjro.20210053

**Published:** 2021-12-17

**Authors:** Shane W. Davy, Diane Bergin

**Affiliations:** 1 Department of Radiology, University Hospital Galway, Galway, Ireland

## Abstract

Osteoporotic vertebral fractures (VFs) are the most common type of osteoporotic fracture. Patients with VF are at increased risk of hip fractures or additional VFs, both of which contribute to patient morbidity and mortality. Early diagnosis of VFs is essential so patients can be prescribed appropriate medical therapy.

Most patients with clinical suspicion for VF have an X-ray of the spine. Many VFs are invisible on X-ray and require further imaging. CT can provide excellent bony detail but uses high doses of ionising radiation. MRI provides excellent soft tissue detail and can distinguish old from new fractures in addition to differentiating osteoporotic VFs from other causes of back pain. Bone scans have a limited role due to poor specificity.

The literature suggests that radiologists frequently miss or do not report VFs when imaging is requested for an alternative clinical indication and when there is no clinical suspicion of VF. Common examples include failure to identify VFs on lateral chest X-rays, sagittal reformats of CT thorax and abdomen, lateral localizers on MRI and scout views on CT.

Failure to diagnose a VF is a missed opportunity to improve management of osteoporosis and reduce risk of further fractures. This article discusses the role of radiographs, CT, MRI and bone scintigraphy in the assessment and recognition of osteoporotic fractures. This article focuses on opportunistic diagnosis of VFs on imaging studies that are performed for other clinical indications. It does not discuss use of dual energy X-ray absorptiometry which is a specific imaging modality for osteoporosis.

## Introduction

In an era of increasing life expectancy, osteoporosis has become a major global health concern.^1^ Osteopororis is a metabolic disease of bone and at least one-third of all post-menopausal females will suffer an osteoporotic fracture in her lifetime.^2^ Global estimates postulate that approximately 20 osteoporotic fractures occur every minute and the National Osteoporosis Foundation (NOF) estimates that approximately 54 million Americans suffer from osteoporosis resulting in 2 million fractures annually.^3^ Many population-based studies have demonstrated concerning upward trends for increasing incidence of hospitalisations for osteoporotic fractures worldwide.^4–6^

Vertebral fractures (VFs) account for up to 50% of osteoporotic fractures making them the most common fracture subtype.^7^ Incidence of VFs increases with increasing age, with significant heterogeneity between sexes, origin and age of onset.^7,8^ Up to 26% of Scandinavian females will be diagnosed with at least one VF in her lifetime.^8^ VFs are a major cause of patient pain, reduced mobility and many patients who have sustained a VF suffer with the psychological fear of isolation and loss of independence.^9,10^ Additionally, sustaining a VF is an independent risk factor for patient mortality, emphasising its significance as a major public health concern.^11^ Studies show that patients with previous VFs are five times more likely to obtain an additional VF and are twice as likely to suffer a hip fracture with resulting morbidity and mortality.^12,13^

Encouragingly, robust evidence has shown that early intervention with pharmacological agents such as bisphosphonates result in a relative risk reduction of up to 0.6 for VFs and up to 0.8 for non-VFs.^14^ Therefore, it is vital that VFs are correctly diagnosed so that patients are appropriately investigated and placed on correct medical therapy. Unfortunately, there is a discrepancy between best recommended management and real-life clinical practice with one study concluding that less than one-quarter of patients diagnosed with an osteoporotic fracture are appropriately investigated and treated for osteoporosis.^15^

Studies suggest that radiologists have contributed to the current problem due to non-diagnosis or underreporting of VFs.^16,17^ VFs are evident on various imaging modalities which are performed for alternative clinical indications but are frequently not reported by radiologists.^18,19^ Use of terminology such as ‘wedging’, ‘endplate compression’ and ‘endplate concavity’ in radiology reports can be confusing and may not be understood to indicate the presence of osteoporotic VF by the reading physician. Non-diagnosis or inappropriate reporting of VFs in this way is a missed opportunity to diagnose osteoporosis, impedes adequate medical treatment and renders the patient vulnerable to sustaining future osteoporotic fractures.^15^

In the current paper, we discuss radiological assessment of VFs and describe how fractures can be diagnosed on the most commonly used imaging modalities, plain film, MRI, CT and bone scans.

### Assessment of fractures

Genant et al devised the semi-quantitative (SQ) method for describing VFs. This method has high inter- and intraobserverer agreement, even amongst inexperienced reviewers.^20^ SQ is relatively straight-forward method to grade fractures and avoids otherwise confusing language which may be misinterpreted. First described on lateral radiographs, SQ method employs visual inspection to grade VFs. Grade 0 is normal without loss of vertebral body height. Grade 0.5 are borderline vertebrae. Grade 1 fractures show mild deformity with approximately 20to 25 % loss of height and 10to 20 % reduction in area. Grade two fractures are moderately deformed with 25–40 % loss of height and 20–40 % loss of area. Grade 3 VFs have lost 40% or more of their height and area.

### Recognition of fractures

#### Imaging modalities

##### Plain films

**Figure 1.**
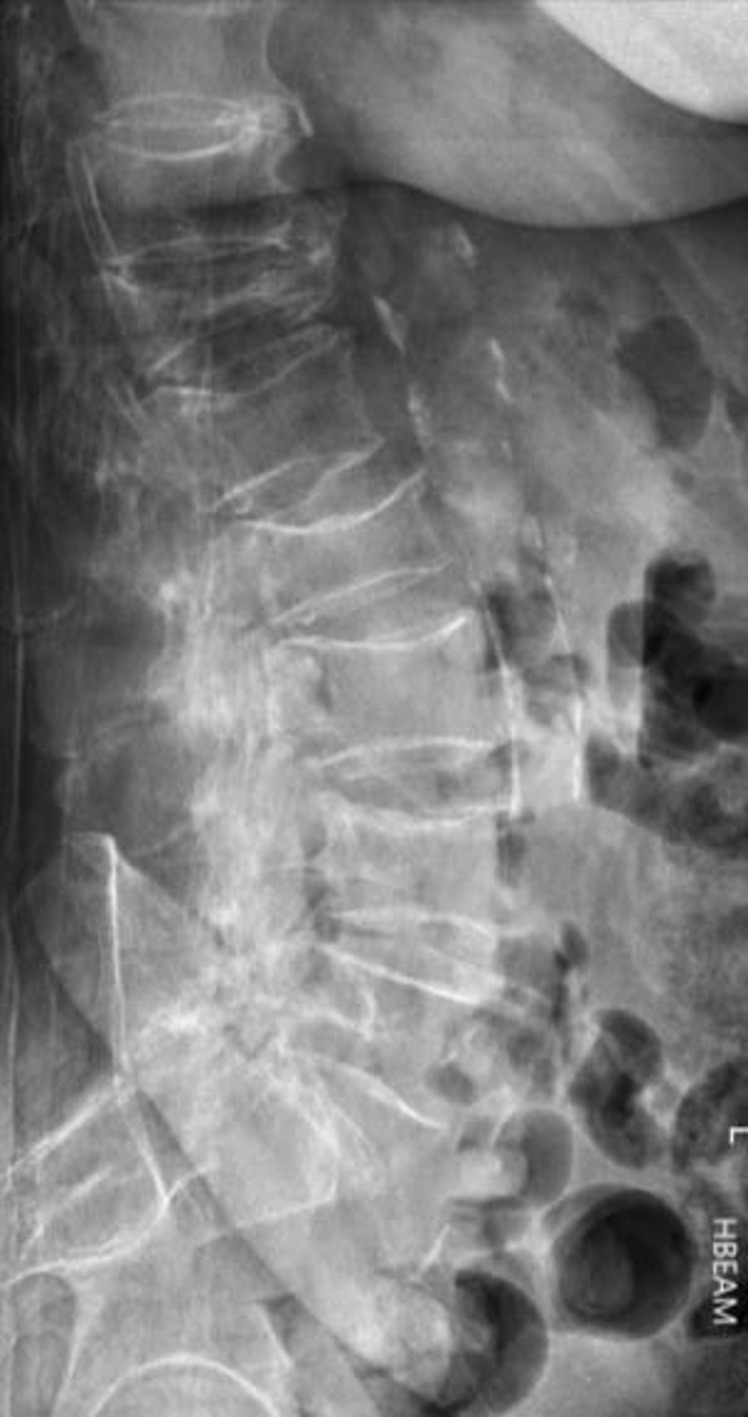
Lateral lumbar spine radiograph of an 80-year-old female with multiple insufficiency compression fractures; severe anterior wedge fracture at T12, mild compression fracture of L1 and L4 superior endplates and moderate compression fracture at L2.

**Figure 2.**
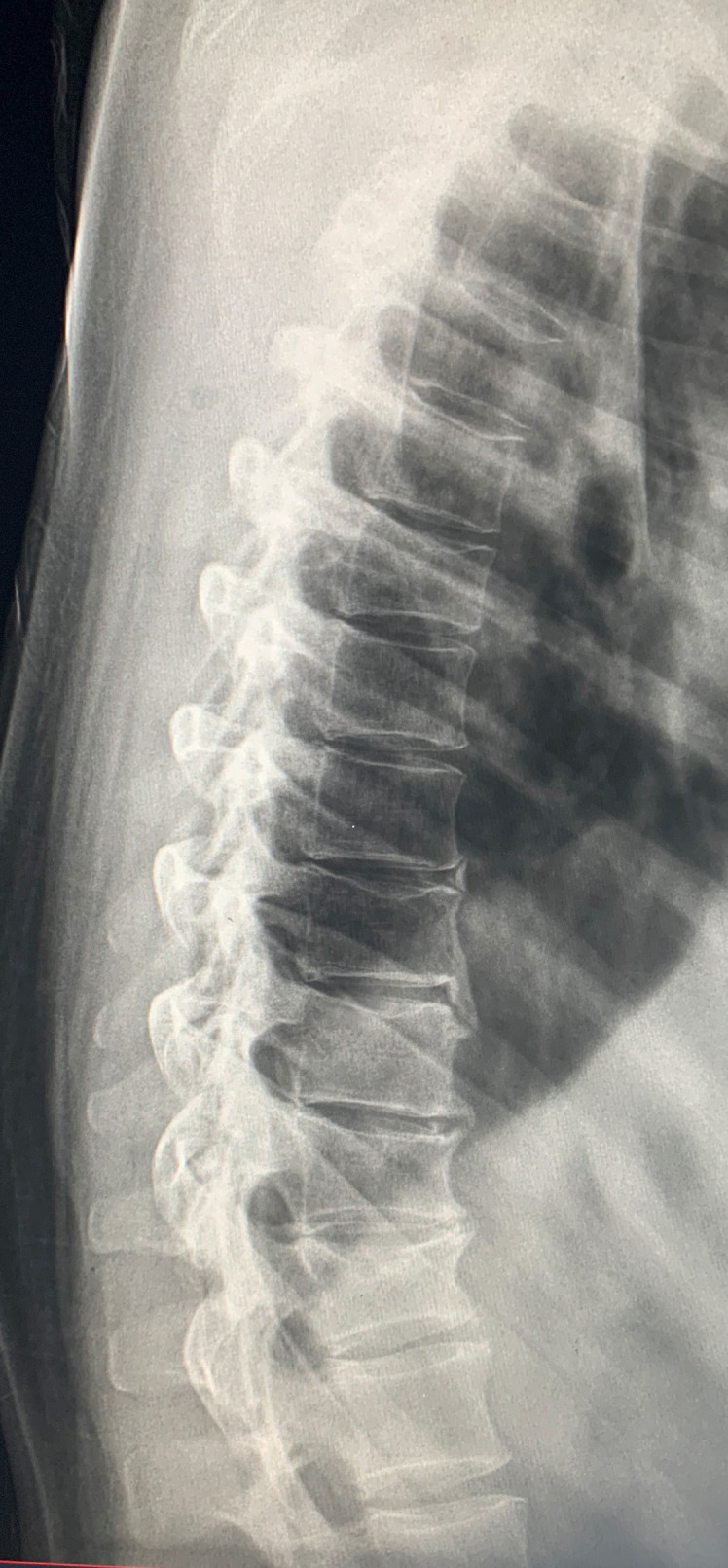
Lateral thoracic spine radiograph with multiple mild vertebral insufficiency compression fractures of T4, T5, T6,T9, T11 and moderate compression fracture of T12.

For clinically suspected VFs, plain films including anteroposterior (AP) and lateral projections are usually the first line of investigation. The lateral film is particularly useful (Figures 1–2). The radiologist should carefully examine the vertebral body outline, especially the superior and inferior endplates to ensure VFs are not missed. The pedicles should be examined for symmetry on the AP film. Subjectively identifying reduced bone density should heighten the index of suspicion for VFs as these patients are at much greater risk.

Dynamic radiographs of the vertebrae can increase the likelihood of correct diagnosis on plain radiography. This method allows the radiologist to compare supine images with lateral sitting radiographs to evaluate for changes in vertebral body height. The sensitivity and specificity of dynamic radiographs for diagnosing acute VFs is 66 and 96%, respectively.^21^ While moderate and severe VFs are rarely misdiagnosed, there are a number of conditions which can be mistaken for mild VFs leading to overdiagnosis. These include developmental short vertebral height, physiological wedging, Scheuermann’s disease, degenerative scoliosis, Schmorl’s nodes and Cupid’s bow deformity (smooth developmental curvature of the inferior endplate of lumbar vertebra).^22^ Possible reasons for underdiagnosis of VFs by non-musculoskeletal radiologists include focusing on other acute findings, lacking specialist knowledge about osteoporosis/osteoporotic VFs or simply ignoring osteoporotic VFs completely.^23^

Vertebrae are included on many plain films when there is no clinical suspicion of VF. Examples include abdominal radiographs for patients with abdominal pain or chest radiographs in patients with cardiorespiratory symptoms. Less commonly, the vertebrae are incidentally imaged during barium investigations, interventional, cardiac and fluoroscopic procedures. Even if not performed to outrule a VF, each imaged vertebra should be carefully evaluated to ensure no underlying occult VF. Despite the obvious opportunity to diagnose VFs in this way, there is a paucity of published literature in the area. The most studied radiographic technique to incidentally diagnose VFs is the chest radiograph. In a large study of over 10,000 post-menopausal females who underwent a lateral chest X-ray, 41% of radiologists who identified a VF failed to document it in the report summary, and only 36% were prescribed bone protection on discharge.^24^ In a smaller retrospective review of chest X-rays of post-menopausal females, Gehlbach showed that 14.1% had a moderate or severe VF visible on chest radiograph.^16^ Unfortunately, less than one-quarter of visible VFs were referenced in the radiologists’ summary, and only one-seventh of these patients received a discharge diagnosis of VF. As a result, only 18% of patients were discharged with appropriate medical therapy for underlying osteoporosis. The lateral chest radiograph on elderly patients is an opportunity to incidentally diagnose VFs by assessing vertebral bodies and clearly reporting them in the final summary.^25^

Despite their importance in initial investigation for suspected VF, many patients with VFs will have no morphological change on plain films. It is important not to dismiss patient symptoms based on normal radiographs since many patients with normal plain films may only have acute changes detectable on MRI.^26^ Loss of vertebral height may not be evident at time of acute symptoms but can be evident on subsequent follow-up radiograph.

## MRI

**Figure 3.**
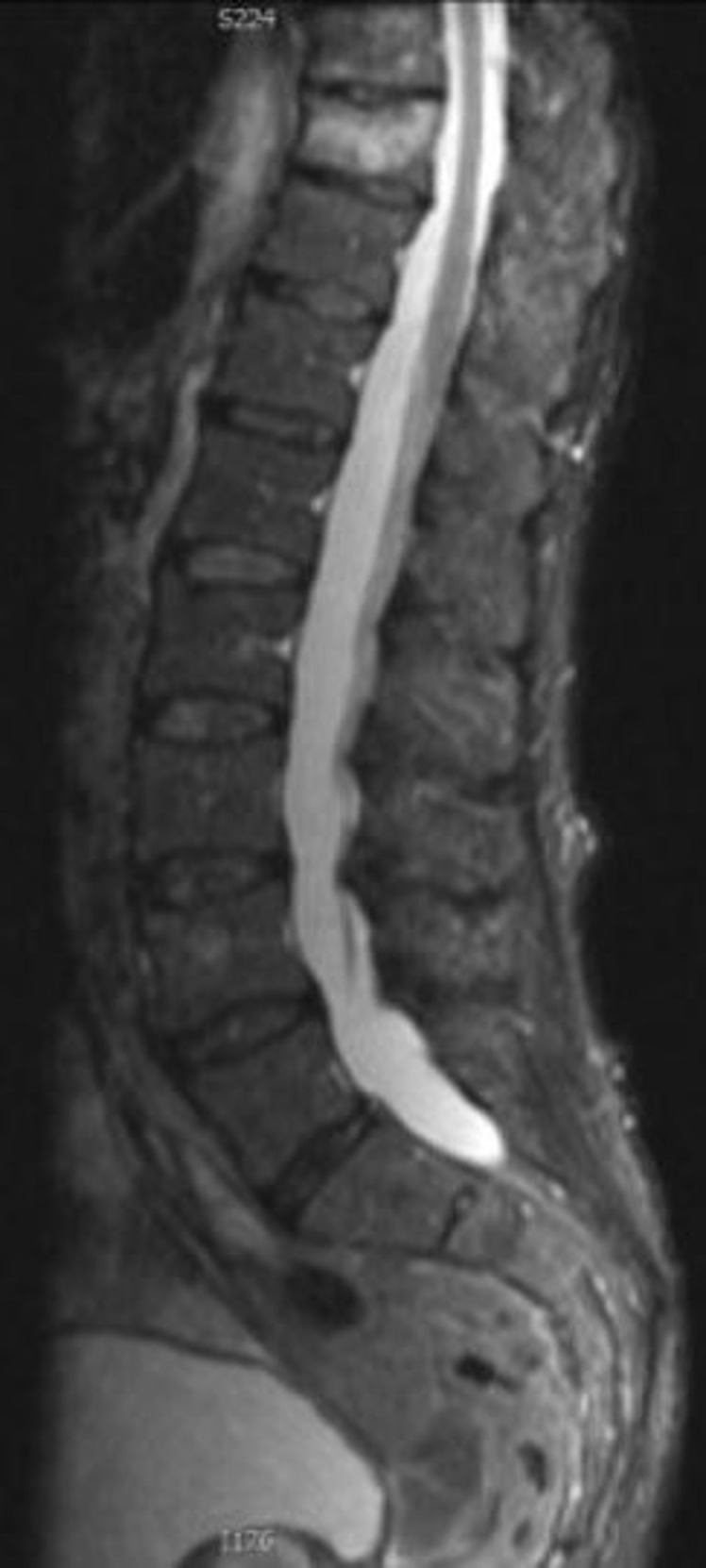
Sagittal STIR image of an acute mild compression osteoporotic fracture of T10 in a 67-year-old female patient. STIR, short tau inversion recovery.

MRI is a time-intensive imaging modality with relative contraindications such as claustrophobia, presence of a non-conditional pacemaker and first trimester of pregnancy. MRI has a sensitivity of 100% in detecting spinal trauma and is an excellent method to diagnose and assess VFs.^26^ MRI has a sensitivity and specificity of up to 82 and 98%, respectively, for distinguishing osteoporotic VFs from other types of fracture,^27^ (Figure 3). In addition to identifying a VF, MRI may also diagnose other uncommon causes for back pain such as infection or malignancy, and also allows assessment of spinal ligaments, spinal cord, surrounding cerebrospinal fluid and meninges.

The short tau inversion recovery (STIR) sequence is particularly useful as it nullifies marrow fat signal over a large body area such as the entire vertebral column. STIR sequences are helpful to differentiate benign osteoporotic VFs from those caused by malignancy.^28^ The presence of marrow oedema recognised as high signal on fluid sensitive STIR or *T*_2_ weighted fat-saturated sequences indicates recent fracture. Marrow oedema is absent in a chronic VF. Benign VFs typically are seen as linear low T1 signal. Malignancy or infection in contrast cause diffuse nonlinear replacement of the normal marrow of the vertebra.

For every MRI study performed, initial localiser sequences are utilised by radiographers to plan image acquisition. These localisers are obtained from thick slices and are not suitable for diagnostic detail but do represent an opportunity to diagnose a VF when not suspected. The importance of reviewing localisers for fractures with strong interobserver agreement to detect VFs in the thoracic and lumbar spine has been reported.^29^ In another study, musculoskeletal radiologists examined 856 localisers of patients undergoing breast MRI. The authors concluded that 8.9% of patients had a VF visible on the MRI localiser, but none were documented in the final report.^30^ MRI localisers are a quick and reliable method of diagnosing VFs when not suspected and may negate the necessity for further imaging or using ionising radiation.

### CT

CT uses high doses of ionising radiation to acquire images. CT imaging is available 24 h in most tertiary hospitals and offers almost instant acquisition of images. CT has excellent sensitivity and specificity for identifying VFs at 100 and 97%, respectively.^31^ CT of the spine may be requested when a VF is clinically suspected and when the radiograph is normal. Of note, a non-displaced fracture accompanied by marked osteopaenia, may not be evident on CT. In patients with known VF, CT can help to provide additional information such as stability of the fracture and protrusion of bone fragments. CT can also aid with clinical decisions such as patient suitability for surgical intervention or vertebroplasty.

**Figure 4.**
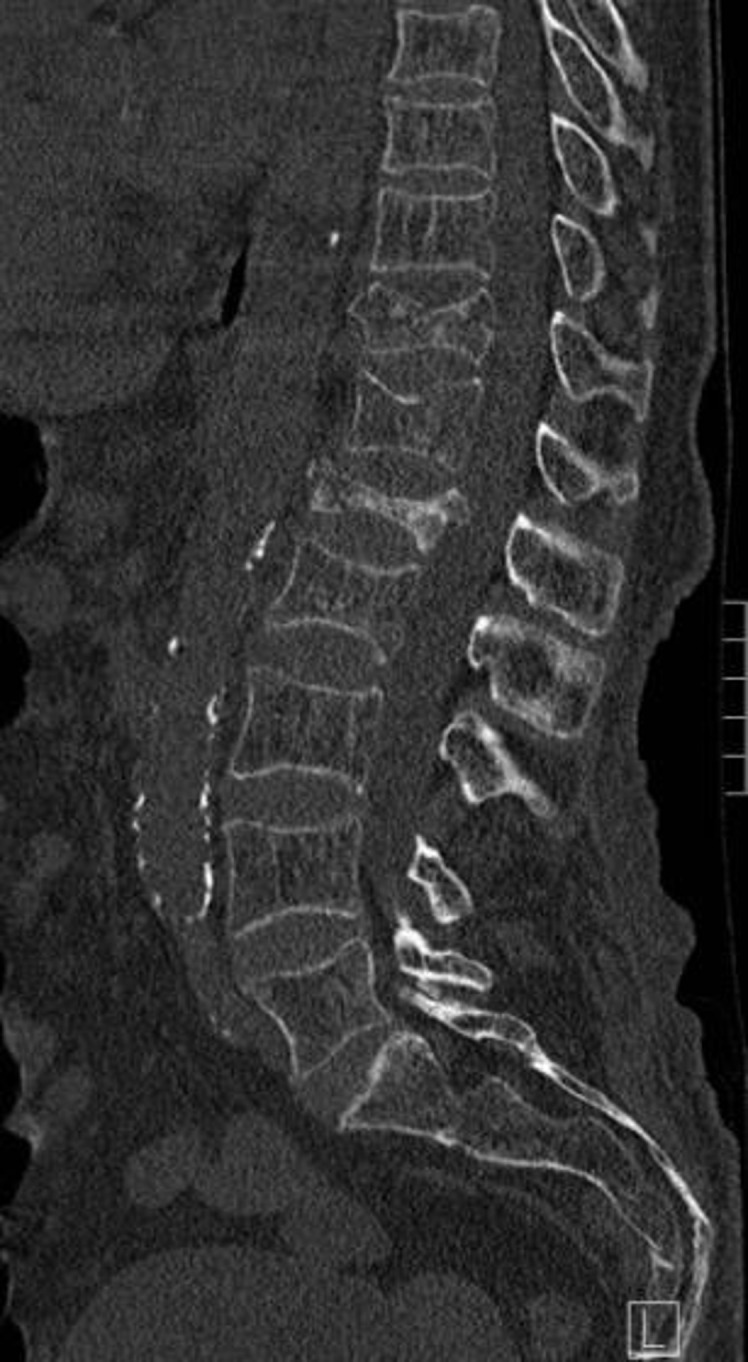
Sagittal reformatted CT of the lumbar spine in an 83-year-old female demonstrating severe osteoporotic compression fracture of L1, moderate compression fracture of T11 and mild compression fracture of L2

The majority of CTs are performed for clinical indications not specifically related to identification of VFs. Examples include cardiac CT, CT pulmonary angiogram and CT thorax to evaluate for thoracic pathology and CT kidneys, ureters and bladder, CT abdomen/pelvis, CT colonography and CT peripheral angiograms/venograms performed to identify intra-abdominal pathology. Vertebral morphology, particularly on sagittal reformats is well visualised on these CT studies. Modern CT scanners can display vertebrae in the region imaged in excellent bony detail in coronal, sagittal and axial reformats without the requirement for further imaging or radiation exposure to the patient. Of these, the sagittal reconstructions are particularly important to diagnose VFs (Figure 4).^32^

Despite the ability to utilise CT to diagnose occult VFs, CT is often not effectively exploited in this way. A New Zealand study retrospectively reviewed sagittal reconstructions of CT abdomen or thorax in patients over 65 years. 22 of 175 patients had a VF visible on sagittal reconstruction, and 77% of these had previously undiagnosed VF. The authors concluded that reviewing reformatted CT of the abdomen and pelvis improved diagnosis of VFs but are frequently not reported—thereby missing an opportunity to treat with appropriate medical therapy to reduce future osteoporotic fractures and associated mortality.^33^

Similar to localisers in MRI, CT scout views are obtained prior to final image acquisition. These use low levels of radiation to acquire two-dimensional images which are used to plan the final CT image. Lateral CT scout views may show fractures not visible on axial CT images. One study of 300 CT scans involving the thoracic and lumbar spines demonstrated the sensitivity and specificity of diagnosing VFs on scout views to be 98.7 and 99.7%, respectively. The authors concluded that scout views should be used to evaluate for VFs on CTs performed for other clinical indications.^34^

### Skeletal scintigraphy (bone scans)

^99m^Tc is a radioisotope which can be bound to methylene diphosphonate (MDP) and injected intravenously. The radioisotope travels through the patient’s bloodstream and binds to remodelling bone. 3 h after injection, patients are placed on a gamma camera which identifies bony hotspots where ^99m^Tc has accumulated. 80% of VFs are visible as hotspots, usually linear in morphology, at 24 h following injury and almost all return to normal within 2 years.^35^

**Figure 5.**
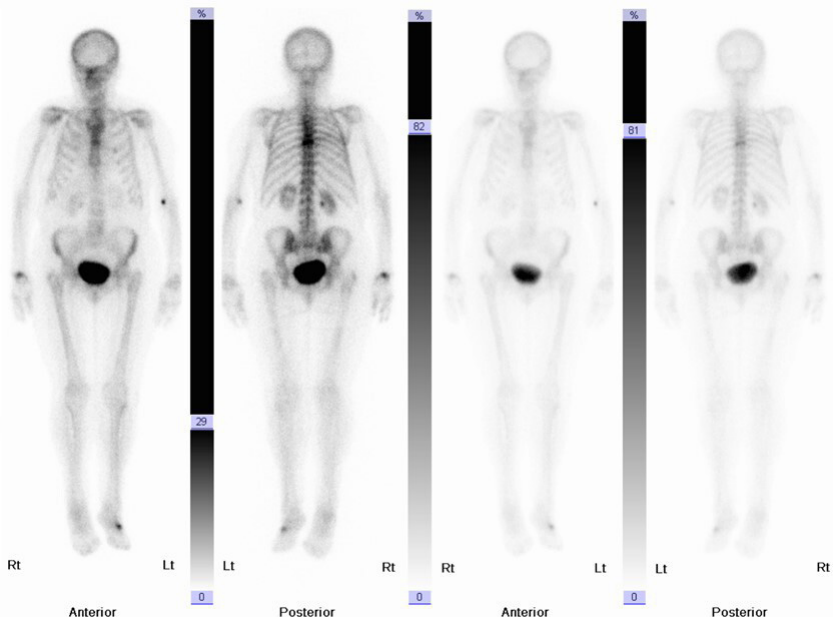
There are foci of increased radioisotope uptake in the mid-thoracic spine on bone scan.

**Figure 6.**
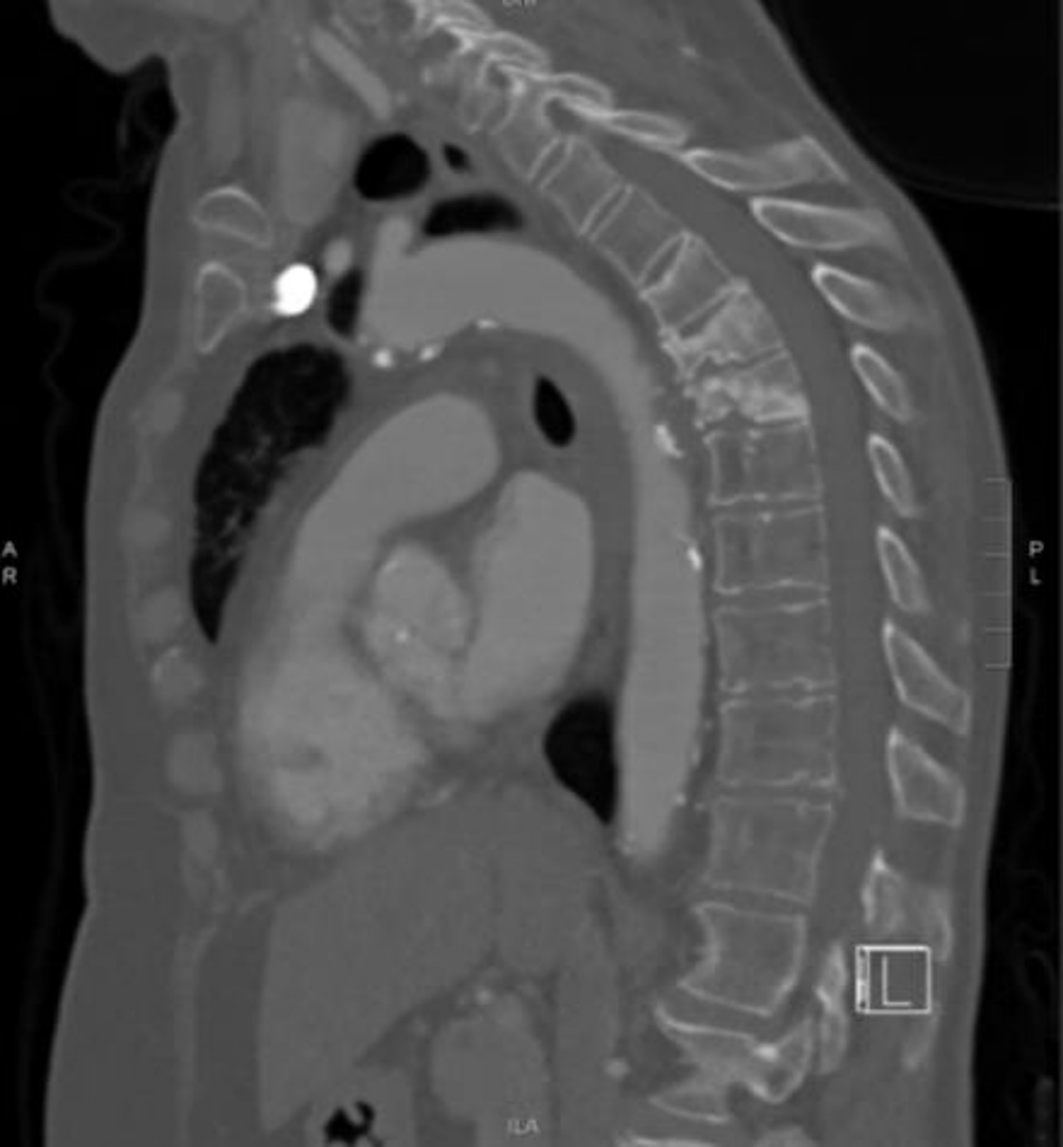
Sclerotic pathological wedge compression fractures of T6 and T7 secondary to metastatic disease on sagittal reconstruction of staging CT thorax in patient with primary non-small cell lung cancer correlating with uptake in bone scan in Figure 5.

The major limitation of bone scans is their poor specificity. The most common indication for performing bone scans is to identify osseous metastatic disease in patients with known primary malignancy. However, bone scans are also utilised to identify occult fractures or osteomyelitis. Due to their non-specific nature, hotspots can also be caused by degenerative changes. Therefore, a nuclear medicine specialist may have diagnostic difficulty in distinguishing the exact cause of a hotspot. For this reason, bone scans (Figure 5) are often reported in conjunction with other available imaging such as MRI, CT (Figure 6) or plain films.

## Discussion

Osteoporosis is an increasing public health concern and predisposes patients to VFs. Prompt diagnosis and early intervention with appropriate medical treatment is imperative. The literature shows a failure by radiologists to report incidental VFs, and many which are diagnosed are never transcribed into the patient discharge summary. Untreated and undiagnosed VFs can significantly impact on a patients quality of life and life expectancy. These patients suffer intolerable pain, lose their independence, suffer psychologically due to fear of isolation, many require polypharmacy for pain control, and all are at substantive risk of future osteoporotic fractures. The mid-thoracic region and thoracolumbar junction are the most commonly affected areas and often lead to spinal kyphotic deformities. Kyphosis predisposes to loss of balance, further muscle fatigue, further degenerative changes at adjacent intervertebral joints, restrictive lung disease, inability to work and loss of earnings.^36^

Unfortunately, many radiologists use equivocal language such as ‘loss of height’ or ‘wedging’ to describe VFs. This terminology is ambiguous for referring physicians and patients and can contribute to inappropriate management of underlying osteoporosis. An alternative strategy, described by Gehlbach et al is the semi-quantitative method for grading VFs. Even amongst inexperienced observers, the SQ method for grading fractures demonstrates high levels of agreement.^16^ Clearly stating the existence of an osteoporotic fracture in this way may improve the percentage of patients being discharged on appropriate medical therapy.

A number of imaging techniques performed for various clinical indications may show VFs in their field of view. However, there is underreporting of VFs which are clearly visible on lateral chest radiographs, MRI localisers and CT scout views. Failure to request bony sagittal reformats from CT scans often renders VFs invisible, even to experienced musculoskeletal radiologists. The term ‘inattentional blindness’ refers to an inability to notice unexpected events when immersed in an alternative task. In one experiment, 83% of expert radiologists failed to recognise a gorilla drawn onto a stack of CT images when they were focusing on finding pulmonary nodules.^37^ Another phenomenon coined ‘satisfaction of search’ refers to a relative difficulty in identifying further pathological findings following identification of another significant abnormality.^38^ Both are relevant to radiologists when searching for potentially life-threathening pathology on X-ray, MRI or CT and VFs can easily be overlooked.

Dedicated education programmes delivered to radiologists and internal medical physicians may help to improve the diagnosis and management of VFs. In one study, recognition of VFs amongst internists almost doubled from 22 to 43% following provision of basic lectures, posters and flyers. The same study demonstrated a significant increase in patients discharged on osteoporosis treatment from 11 to 40%.^39^ In another study, there was a marked improvement in the ability of a radiology resident to correctly identify VFs after undergoing specific teaching.^40^

## Conclusion

In conclusion, VFs are a major health concern in an era of aging population. Many factors have contributed to poor diagnosis and treatment of VFs. When identified, ambiguous terminology should be avoided and SQ method employed. Irrespective of clinical indication or imaging modality, a high index of suspicion for VFs should be employed at all times. Basic education programmes delivered to radiologists and internists have been shown to improve diagnosis and treatment of VFs.
